# Facile Preparation of Stable Solid-State Carbon Quantum Dots with Multi-Peak Emission

**DOI:** 10.3390/nano10020303

**Published:** 2020-02-10

**Authors:** Yanning Zheng, Jingxia Zheng, Junli Wang, Yongzhen Yang, Taiping Lu, Xuguang Liu

**Affiliations:** 1 Key Laboratory of Interface Science and Engineering in Advanced Materials, Taiyuan University of Technology, Ministry of Education, Taiyuan 030024, China; tyutzyn@163.com (Y.Z.); zhengjingxia@tyut.edu.cn (J.Z.); wangjunli01@126.com (J.W.); liuxuguang@tyut.edu.cn (X.L.); 2 Institute for New Carbon Materials, Taiyuan University of Technology, Taiyuan 030024, China

**Keywords:** solid-state luminescence, multi-peak emission, yellow carbon quantum dots, white light-emitting diode

## Abstract

Aggregation-caused quenching (ACQ) effect, known as the main cause to restrain solid-state luminescence of carbon quantum dots (CQDs), hinders further application of CQDs in white light-emitting diodes (WLED). Here, a complex of CQDs and phthalimide crystals (CQDs/PC) was prepared through a one-step solvothermal method. CQDs/PC prevented CQDs from touching directly by embedding the CQDs in phthalimide crystal matrix in situ, which effectively reduced the ACQ effect. Furthermore, CQDs/PC exhibited multi-peak fluorescence spectra that span the green, yellow and orange spectral regions. Finally, a WLED fabricated based on CQDs/PC achieved a color-rendering index of 82 and a correlated color temperature of 5430 K. This work provides a quick and effective strategy to apply CQDs to WLED.

## 1. Introduction

Carbon quantum dots (CQDs) have attracted widespread attention recently in optoelectronic applications owing to their advantages such as being abundant raw materials, their environmental-friendliness, and their low toxicity [1,2]. The graphite-like carbon core and surface state endue them with excellent optical performance and adjustable fluorescence [2,3]. Because their quantum yield (QY) is comparable to commercial phosphors [4,5,6,7], CQDs have great potential to be used in phosphors for white light-emitting diodes (WLED) [8,9,10,11]. However, there are two major bottlenecks towards applying them to WLED on a large-scale. First, the aggregation-caused quenching (ACQ) effect is the primary obstacle that should be eliminated for CQDs-based phosphors [12]. Second, the single-peak emission of most CQDs is another hindrance that should be settled for fabrication of a high color-rendering index (CRI) WLED [13,14,15,16,17].

With respect to ACQ, it is usually attributed to resonance energy transfer or π–π interaction induced by CQDs touching [12,18]. The general approaches to realize solid-state fluorescence CQDs are dispersing them in matrices, such as starch, polyvinyl alcohol, or polymethyl methacrylate [7,19,20,21,22]. Nevertheless, most of these solutions need a secondary treatment to the obtained CQDs. For simplifying the process, two groups have proposed a one-step method to efficiently prepare solid-state luminescence CQDs in one synthesis procedure [23,24]. However, it still requires complicated purification and post-treatment, such as centrifugation and dialysis.

Solid-state luminescence CQDs can be prepared by a simple one-step method, but their single-peak emission is still a hindrance to realize high CRI WLED. In general, high CRI requires the luminescence spectrum of WLED to exhibit moderate intensity in each region of visible light [25]. Therefore, the most commonly used strategy is the composition of multi-color CQDs obtained through multiple synthesis processes [21,26,27,28]. However, this complicated strategy is not conducive to preparing CQDs-based high-CRI WLED quickly and efficiently. In recent works, a simplified process derived from a one-step method was proposed to realize solid-state luminescence CQDs with broad emission spectra [29,30]. Although such products have been used to fabricate high-CRI WLED, complicated purification and post-treatment were needed in these methods.

In this paper, a one-step method, which has simple purification or post-treatment procedures, is proposed to prepare solid fluorescent CQDs with multi-peak emission. Phthalic acid and formamide were used to synthesize a solid fluorescent composite integrating CQDs and phthalimide crystals (CQDs/PC). CQDs/PC prevents ACQ effect by embedding CQDs into phthalimide crystal in situ. On account of the macroscopic size, the initial product of CQDs/PC only needs the post-treatment of filtration and drying. Multi-peak emission that covers the green, yellow and orange spectral regions and remarkable thermal stability make CQDs/PC suitable for high-CRI WLED. The proposed CQDs/PC provide a quick and effective strategy to apply CQDs to WLED.

## 2. Materials and Methods 

### 2.1. Chemicals and Materials

Phthalic acid was purchased from Tianjin Guangfu Fine Chemical Research Institute (Tianjin, China). Formamide was acquired from Tianjin Fengchuan Chemical Reagent Co., Ltd. (Tianjin, China). N, N-Dimethylformamide (DMF) was provided from Tianjin Tianli Chemical Reagent Co., Ltd. (Tianjin, China). Glycerol OE-625 silicone LED encapsulation AB glue was purchased from Dow Corning Co., Ltd. (Tokyo, Japan). All chemicals were commercially available and used without further purification.

### 2.2. Synthesis of CQDs/PC

Phthalic acid (1.6 g) was dissolved in 20 mL of mixed solvent of formamide and glycerol with a volume ratio of 1:1. The mixture was then transferred into a 40 mL Teflon stainless steel autoclave and reacted in an oven at 453 K for 4 h. After cooling to room temperature, the solid–liquid mixture product was filtered through an organic filtration membrane (0.22 μm) to get yellow acicular solid. Finally, yellow powder of CQDs/PC was obtained after drying and grinding.

**Figure 1:**
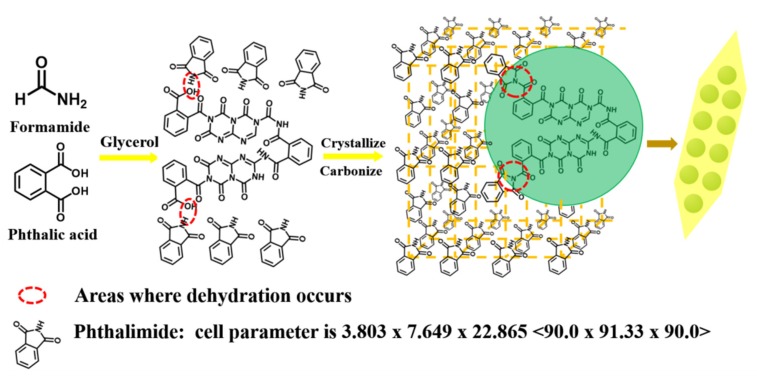
Synthesis principle of carbon quantum dots (CQDs) and phthalimide crystals (CQDs/PC).

The synthesis principle of CQDs/PC can be illustrated as Figure 1. Since phthalic acid acts not only as a carbon source, but also as a reactant to react with amino compounds to generate phthalimide, the reaction system of phthalic acid, formamide and glycerol can produce the precursor of CQDs and phthalimide molecules as the solvothermal reaction proceeds. Free CQD precursors, with unreacted carboxyl groups on their surface, keep colliding with phthalimide molecules. Consequently, the carboxyl groups of CQD precursors are dehydrated and condensed with the imide groups of phthalimide. Afterwards, phthalimide begins to nucleate owing to the supersaturation of phthalimide molecules in the solvent system. As the phthalimide crystal nuclei grow, CQD precursors are constantly adsorbed and embedded into phthalimide crystals following their carbonization. Finally, the composite structure of CQDs–phthalimide crystals grows into the same needle-shape crystals as phthalimide. CQDs are dispersed in phthalimide crystals to prevent aggregation and realize solid-state luminescence.

### 2.3. Fabrication of WLED

CQDs/PC phosphor (50 mg) was mixed evenly with 0.2 mL of A-gel, and then 0.2 mL of B-gel was added (the volume ratio of A to B is 1:1). The mixture of CQDs/PC and AB glue was uniformly coated on an LED lampshade and assembled with 460 nm blue LED chip after solidification.

### 2.4. Characterization

Transmission electron microscopy (TEM) and high-resolution transmission electron microscopy (HRTEM) images of CQDs/PC phosphor were obtained on JEOL JEM-2010. The powder sample was first dispersed in deionized water ultrasonically and then dripped onto ultra-thin carbon films. Fourier-transform infrared (FTIR) spectra were obtained on a Bruker Tensor 27 spectrometer with the sample in KBr disk. The X-ray diffraction (XRD) pattern of the CQDs/PC was measured by Rigaku-D/MAX 2500 diffractometer with Cu Kα (λ = 1.5406 Å) radiation at a scanning speed of 4°/min in the 2θ range from 10° to 80°. Photoluminescence (PL) spectra were recorded on Horiba Fluoromax-4 fluorescence spectrophotometer. The thermogravimetric (TG) curve was characterized by Setaram Labsys Evo TG analyzer under a high-purity argon atmosphere with a 10 K/min heating rate. The absolute QY of CQDs/PC phosphor was measured by an integrating sphere. X-ray photoelectron spectroscopy (XPS) measurement was conducted on Kratos AXIS ULTRA DLD X-ray photoelectron spectrometer with mono X-ray source Al Kα excitation. WLED devices were tested by the F-star photoelectric testing system.

## 3. Results and Discussion

### 3.1. Morphology and Structures

**Figure 2:**
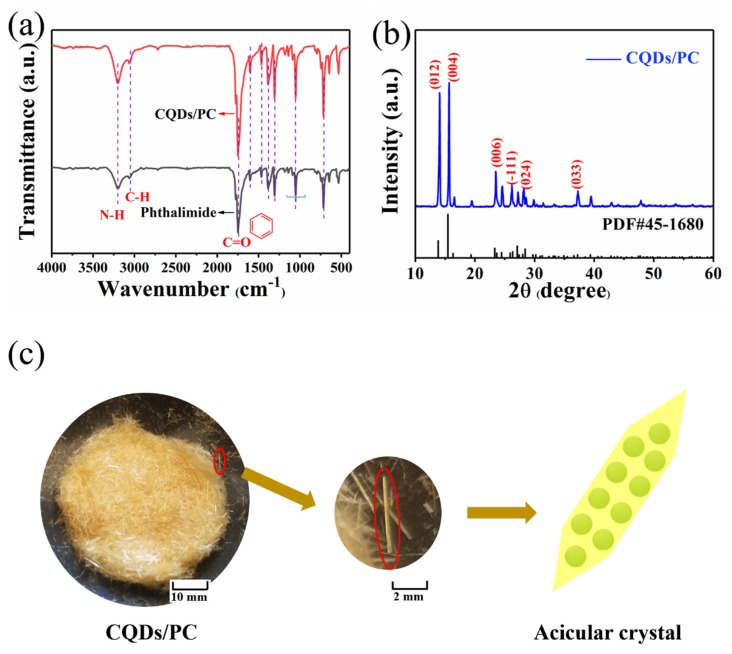
Fourier-transform infrared (FTIR) spectra of (**a**) CQDs/PC and phthalimide. (**b**) X-ray diffraction (XRD) patterns of CQDs/PC and standard phthalimide. (**c**) Low and high magnification physical photographs of CQDs/PC, and model diagram of their microstructure.

The structure and composition of CQDs/PC synthesized by one-step solvothermal method was investigated via FTIR and XRD. As shown in Figure 2a, the FTIR spectra of CQDs/PC and phthalimide have almost identical patterns and band positions. In the spectra, the broad absorption band at 3202 cm^−1^ is assigned to the stretching vibrations of N-H and the strong band at 1749 cm^−1^ is corresponding to the vibrations of C=O. As for the absorption bands at 1605–1307 cm^−1^, they are attributed to the vibrations of benzene rings. It can be inferred that the matrix of CQDs/PC is phthalimide crystal. As shown in Figure 2b, XRD patterns of CQDs/PC match well to standard PDF card data of phthalimide crystals. Meanwhile, the narrow full width at half maximum of peaks in XRD patterns indicates that phthalimide has a quite high crystallinity, which corresponds to the outward of CQDs/PC showing a needle shape (Figure 2c). Consequently, it can be inferred that the fluorescent centers emitting yellow fluorescence under UV lamp are embedded in the phthalimide crystal matrix which is illustrated in the diagram in Figure 2c.

**Figure 3:**
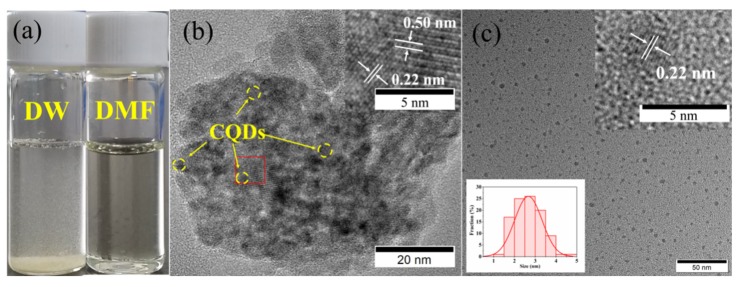
(**a**) Solubility comparison of CQDs/PC in deionized water (DW) and N, N-Dimethylformamide (DMF). (**b**) Transmission electron microscopy (TEM) and high-resolution transmission electron microscopy (HRTEM) images (inset) of CQDs/PC. (**c**) TEM image, the statistical histogram of particle size (the inset on the bottom left corner), and HRTEM image (the inset on the top right corner) of CQDs.

To further explore the inside of CQDs/PC, the TEM images were measured. Figure 3a shows that CQDs/PC is insoluble in deionized water, but soluble in DMF. The TEM image of CQDs/PC dispersed by deionized water (Figure 3b) shows a complex lattice fringes with different spacings. The spacing of 0.50 nm (see HRTEM image in the inset of Figure 3b) corresponds to the (004) planes of phthalimide crystal and the spacing of 0.22 nm corresponds to the (100) planes of graphite [31], which implies that CQDs are embed in phthalimide crystal matrix. Furthermore, the TEM image of CQDs/PC dissolved in DMF (Figure 3c) confirms that the spherical CQDs with spacing of 0.22 nm (see HRTEM image in inset of Figure 3c) and average particle size of 2.7 nm are contained in phthalimide crystals. This shows that CQDs are confined in phthalimide crystals and separated from each other. This structure increases the steric hindrance between CQDs and prevents the quenching effect caused by aggregation.

**Figure 4:**
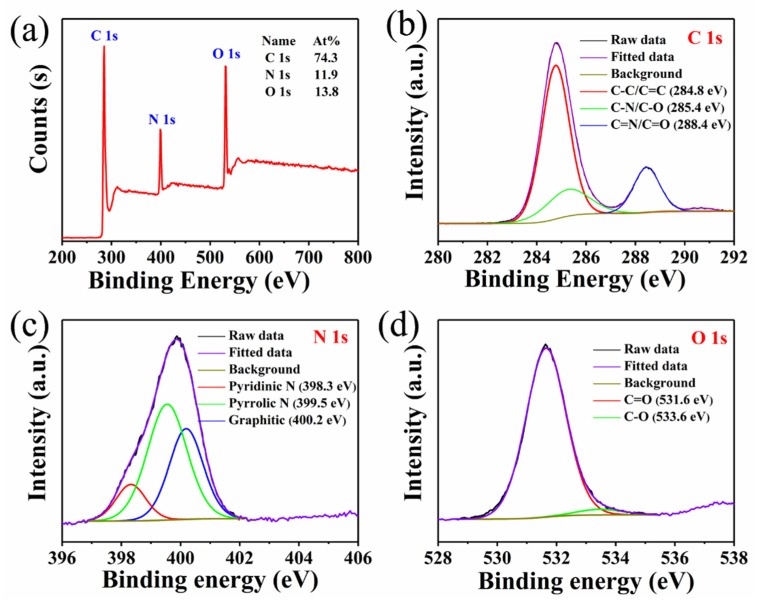
(**a**) X-ray photoelectron spectroscopy (XPS) survey spectra and high-resolution spectra of (**b**) C 1s (**c**), N 1s and (**d**) O ls of CQDs/PC phosphor.

The composition and structure of CQDs/PC phosphor were further determined by XPS. From Figure 4a, the XPS survey spectra show that the element content of C, N and O in CQDs/PC phosphor is 74.3%, 11.9% and 13.8%, respectively. Compared with the element content of C, N and O in phthalimide crystals (67.6%, 9.9%, 22.5%), the content of C and N are increased in CQDs/PC, which is caused by the existence of CQDs. In the high-resolution C 1s spectra (Figure 4b), the three peaks at 284.8, 285.4 and 288.4 eV correspond to C-C/C=C, C-N/C-O and C=N/C=O, respectively [32,33]. The three peaks in the N 1s spectrum (Figure 4c) indicate that the N atoms in CQDs/PC phosphor are mainly in the forms of pyridine N (398.3 eV), pyrrole N (399.5 eV) and graphite N (400.2 eV) [34]. Because pyrrole N is the main form of the N atom in phthalimide crystal, pyridine N and graphitic N are concluded to be the main forms of N in CQDs dispersed in phthalimide crystal. As the only N source in the reaction system, formamide participates simultaneously in the synthesis of phthalimide and CQDs. The high-resolution O 1s spectra (Figure 4d) show that most of O in CQDs/PC phosphor exists in the form of C=O (531.6 eV), while C-O (533.6 eV) only accounts for a tiny proportion [32,34]. It can be deduced that there is scarcely any hydroxyl (-OH) or carboxyl (-COOH) in CQDs/PC.

### 3.2. Optical Properties and Thermal Stability

**Figure 5:**
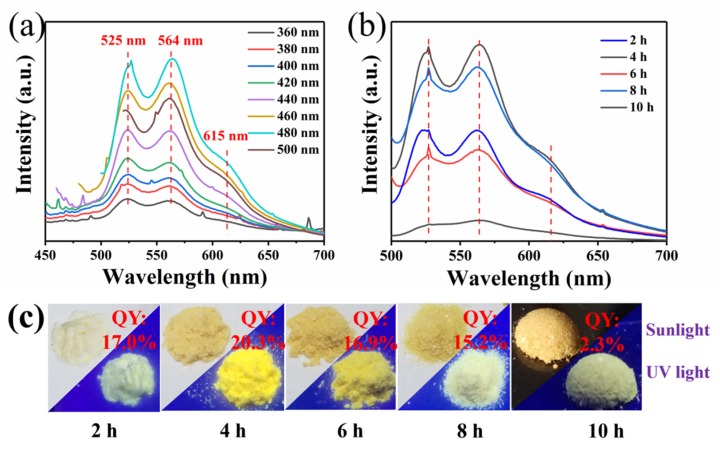
(**a**) Photoluminescence (PL) spectra of CQDs/PC phosphor under different excitation wavelengths ranging from 360 to 500 nm, (**b**) PL spectra of the phosphors prepared with different reaction times at 480 nm excitation light and (**c**) photographs of the CQDs/PC phosphors prepared at different reaction times (2, 4, 6, 8 and 10 h) under day light (above) and 365 nm UV light (below) and their quantum yields (QY).

The optical properties of CQDs/PC are presented in Figure 5. The PL spectra in Figure 5a show three emission peaks at 525, 564 and 615 nm, suggesting the emission spectra of CQDs/PC cover the green, yellow and even orange regions of the visible spectrum. Based on currently existing reports [35,36,37], it can be inferred that the three emission peaks of CQDs/PC derived from three defect states caused by C=O, pyrrolic N and C-N (graphitic N), respectively, on the interface of the CQDs and phthalimide crystal. For WLED, such a broad spectrum is generally corresponding to high CRI, which shows the potential of CQDs/PC phosphor as a single matrix phosphor for WLED. Meanwhile, it can be seen from PL spectra that the positions of the emission peaks of CQDs/PC remain unchanged when excited by different excitation lights. Furthermore, the PL spectra of the samples prepared at 453 K for different times (2, 4, 6, 8 and 10 h) were measured under 480 nm excitation light (Figure 5b, c). It is found that the PL spectra of these samples all display three emission peaks and the positions of the emission peaks remain almost unchanged, which indicates that these products have stable emission centers under different reaction times. The highest absolute QY of CQDs/PC (4 h) was measured to be 20.3% (Figure 5c).

**Figure 6:**
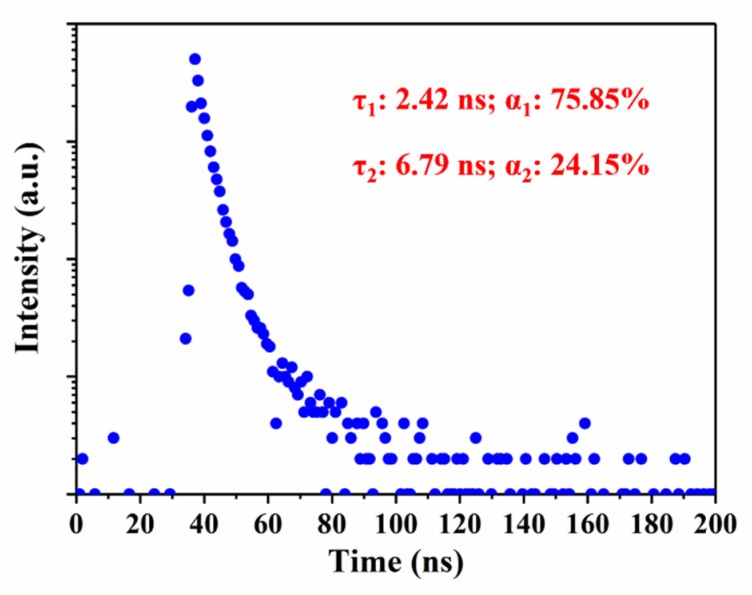
Fluorescence decay profile of CQDs/PC.

To give more insights into the luminescence mechanism of CQDs/PC, the fluorescence decay profile was measured (Figure 6) and it shows a biexponential decay characteristic after fitting Equation (1),

R(t) = α_1_exp(−t/τ_1_) + α_2_exp(−t/τ_2_) (1)

τ_ave_ = (α_1_τ_1_^2^ + α_2_τ_2_^2^)/(α_1_τ_1_ + α_2_τ_2_) (2)

where R(t) is the sum of the individual exponential decay intensities, τ_1_ and τ_2_ are the decay times corresponding to the two individual decay models, and α_1_ and α_2_ are the proportional coefficients of decay time τ_1_ and τ_2_, respectively. τ_ave_ is average lifetime. Here, τ_ave_ is calculated to be 4.48 ns according to Equation (2).

The biexponential decay means that CQDs/PC contains two obvious fluorescent centers, which assigns to the defect states caused by C=O and pyrrolic N [38]. The proportion of τ_1_ (that is α_1_) is 75.85%, which plays a leading role in the radiation lifetime, corresponding to the rich content of C=O in CQDs/PC.

**Figure 7:**
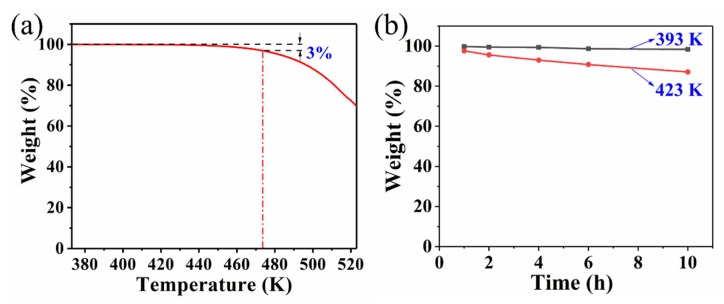
(**a**) Thermogravimetric (TG) curve of CQDs/PC phosphor. (**b**) Residual weight of CQDs/PC phosphor after keeping at 393 and 423 K with different times in air.

To explore the potential application in WLED, the thermal stability of CQDs/PC was investigated. From the TG curve in Figure 7a, the tolerance of CQDs/PC to a certain temperature can be observed. The result of the TG test shows that the weight loss of CQDs/PC is less than 3% before 473 K. The effect of temperature on the stability of CQDs/PC was also investigated by keeping the samples at 393 and 423 K separately for 1, 2, 4, 6 and 10 h, and the ratio of remaining weight of the samples is shown in Figure 7b, which illustrates the good thermal stability of CQDs/PC. This may benefit from the stable chemical environment constructed by phthalimide crystal.

**Figure 8:**
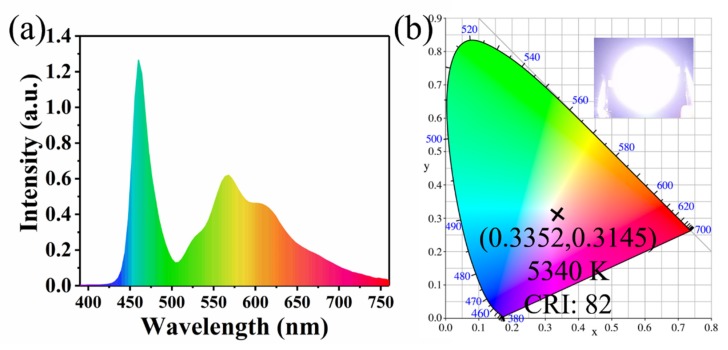
(**a**) Luminescence spectra and (**b**) Commission Internationale de l’Eclairage (CIE) coordinates with the photo (inset) of CQDs/PC phosphor WLED measured at 3.0 V voltage.

Owing to its multi-peak emission and good thermal stability, CQDs/PC is suitable for WLED with high CRI. CQDs/PC was coated on the lampshade of blue-light LED chip to fabricate a WLED device. Figure 8a shows the luminescence spectra of the WLED device under work condition, wherein the emission peak at 460 nm is attributed to the LED chip and the emission peaks at 525, 564 and 615 nm originate from CQDs/PC. From Figure 8b, it can be seen that the Commission Internationale de l’Eclairage (CIE) coordinates of the WLED device are (0.3352, 0.3145), the correlation color temperature (CCT) is 5340 K and CRI is 82. These results show that CQDs/PC phosphor with multi-peak emission can be applied in WLED as a single phosphor.

## 4. Conclusions

In summary, a solid-state fluorescence and multi-peak emission CQDs/PC was prepared by a one-step solvothermal method which requires merely the post-treatment of filtration and drying. Interestingly, CQDs/PC exhibited a CQDs–phthalimide crystal composite structure, which avoids the ACQ effect by embedding CQDs in phthalimide crystals. CQDs/PC shows a multi-peak emission at 525, 564 and 615 nm, and good thermal stability, which is beneficial to fabricating WLED with high CRI. Consequently, CQDs/PC phosphor was used as a single matrix phosphor to combine with a blue LED chip to fabricate WLED devices with CIE coordinates of (0.3352, 0.3145), CCT of 5340 K and CRI of 82, which indicates the potential application of CQDs/PC phosphor in the lighting field.
